# Attitudes of Young Adults toward Animals—The Case of High School Students in Belgium and The Netherlands

**DOI:** 10.3390/ani9030088

**Published:** 2019-03-11

**Authors:** Pim Martens, Camille Hansart, Bingtao Su

**Affiliations:** International Centre for Integrated assessment and Sustainable development (ICIS), Maastricht University, P.O. Box 616, 6200 MD Maastricht, The Netherlands; c.hansart@alumni.maastrichtuniversity.nl (C.H.); su_bingtao@126.com (B.S.)

**Keywords:** animal welfare, young adult, animal attitudes

## Abstract

**Simple Summary:**

Young adults’ attitudes towards animals will be influenced by a number of factors, including: sex, age, nationality/ethnicity, residence area, animal-related activities and hobbies, food habits, culture/religion, education and pet ownership. A case study of Dutch and Belgian high school students shows that levels of concern for animal welfare were distinctly higher among female participants, those who ate little to no meat, Belgian students, pet owners and those who had been to a zoo at least once. In general, students who reported having more contact with animals also had more positive attitudes towards animals.

**Abstract:**

The social context and culture in which individuals grow shapes their perspectives through life. Early on, children learn about animals through storybooks, animated movies, toys, and through interactions with pets and wildlife, and will slowly start to build beliefs around those experiences. Their attitudes towards animals will be influenced by a number of factors, including: sex, age, nationality/ethnicity, residence area, animal-related activities and hobbies, food habits, culture/religion education, and pet ownership. A case study of Dutch and Belgian high school students (aged 12–21) investigated the attitudes of young people towards animals. By using the Animal Attitude Scale (AAS) and the Animal Issue Scale (AIS) questionnaires, our study shows that levels of concern for animal welfare were distinctly higher among: female participants; those who ate little to no meat; Belgian students; pet owners; and those who had been to a zoo at least once. In general, students who reported having more contact with animals also had more positive attitudes towards animals. To understand younger generations and their attitudes toward animals is to understand how future generations will look towards and treat our fellow animals, with which we share the planet Earth.

## 1. Introduction

Animals have accompanied humans for thousands of years, with a strong bond forged between humans and other species. Our relationships with animals can take different forms. On the one hand, animals can serve instrumental purposes: we currently use animals for clothing, for testing a range of human products, for gaining basic insights into human biology and behavior, and as food. On the other hand, human–animal relations are social. The clearest example is the practice of pet-keeping, with people attributing a special status to their pets [1].

Studies have shown that most children reject the idea of humans being animals [2], although they do have a propensity to anthropomorphise animals [3,4]. Most children have an appreciation for animals on the emotional and recreational levels. They tend to show affection as well as concern for them, in contrast to the more practical and utility-based perspectives of adults [3,5]. Kellert [3] found that children were strongly emotionally attached to individual animals. Hunting was not popular among the children and was only deemed acceptable if the end purpose was to feed oneself as opposed to sheer trophy hunting. Similar results were found by Pagani, et al. [6], who reported the majority of children were against hunting, zoos, animals used in circuses, and their exploitation for leather. Slight preferences were expressed for zoos compared to circuses, perhaps because zoos pursued a greater mission in terms of education and conservation. Earlier, Driscoll [7] outlined the different views adults have on how humans should use animals. Despite considerable opposition, most were in favour of animals being used in medical or scientific research, but did not approve of their use in product testing. Expressing similar ethical concerns, a large proportion of children disapproved of the use of animals in fur farming [3]. 

Understanding the attitudes that younger generations have toward animals may help us to understand the sustainability of future societies, as our attitude towards animals are central in the sustainability debate. Many factors including gender, age, nationality/ethnicity, residence area, animal-related activities and hobbies, food habits, culture/religion, education, and pet ownership are associated with people’s attitudes toward animals [8,9]. The present study was conducted among high school students in Belgium and the Netherlands. Through this study, we aim to find out whether the variables we mentioned above and other variables like household, house type, meat-eating frequency correlate with young adults’ attitudes toward animals. 

## 2. Methodology

Research into children’s attitudes toward animals in the Netherlands and Belgium was conducted between May and July in 2016. During this period, a paper-based questionnaire was implemented in four different schools, including three schools in the French-speaking province of Walloon, Collège du Christ Roi (N = 54), Athénée Maurice Carême (N = 148), Paul Delvaux (N = 45) and one school in the south of the Netherlands, Rombouts College (N = 120). All participants were high school students aged 12 to 21. Selection of participants in this study was made through simple random sampling; however, only those classes that replied to the invitation to participate in the research are represented here. Schools were contacted via mail and/or telephone prior to visits.

### 2.1. Questionnaire

The questionnaire consisted of four parts. In the first, we asked respondents to provide their demographic details, including age, gender, nationality, highest level of education, household composition, residence area, type of house, presence of a garden, zoo/aquarium visiting frequency, meat-eating frequency, pet ownership and religious affiliation. 

In the second part, we introduced the Animal Attitude Scale (AAS) [10], which was used to assess the participant’s attitude toward animals by means of a Likert scale. The questionnaire consisted of 20 questions rated from 1 (strongly agree) to 5 (strongly disagree) for all questions except questions number: 1,3,4,7,10,11,17,19, 20, which were all reverse coded from 1 (strongly disagree) to 5 (strongly agree). Total AAS scores were calculated by adding up all individual questions scores. Higher AAS scores indicated a higher concern and respect for animals. Questions included: “I do not think that there is anything wrong with using animal in medical research” Or “Wild animals, such as mink and raccoons, should not be trapped and their skins made into fur coats”.

In the third part of the questionnaire, the Animal Issue Scale (AIS) [11] was introduced and served as a complement to the AAS questionnaire. This scale was much longer, comprised of 43 total questions. These questions were grouped across eight separate sections (use of animals, disrupting animal integrity, killing animals, compromising animal welfare, experimenting on animals, changing animals’ genotypes, animals and the environment, and societal attitudes toward animals). Again, respondents had to rate the questions on a five-mark scale ranging from high acceptability to high non-acceptability. Total AIS scores were calculated by adding individual section scores. Akin to the AAS questionnaire, a higher total AIS score indicates a more positive attitude toward animals, and lower acceptability for the issues described [12]. For example, these issues included: “Keeping animals for the education of the public in zoos, wildlife parks, etc.” and “Killing young animals that are dependent on their parents”. 

### 2.2. Statistical Analysis

Responses from the questionnaires were analysed through IBM SPSS Statistics (version 21, Armonk, NY: IBM Corp). Descriptive statistics were used to analyse the independent variables of age, sex, education level, household composition, residence place, housing type, presence of a garden, zoo and aquarium visiting frequency, meat-eating frequency, pet ownership and religious affiliation. Data was then analysed to check for normality so as to use the best fitted statistical test for analysis. All variables were non-normally distributed. They were thus analysed using a Mann–Whitney U-test and a Kruskal–Wallis test. Furthermore, post hoc analyses on the Kruskal–Wallis tests were conducted to calculate the differences between the average scores of each group. All results are assessed for significance based on the cut-off value of 0.05. Similar shapes of distribution were assumed for the Mann–Whitney U-test for all variables. 

## 3. Results

### 3.1. Demographics

In total, 367 hard copy questionnaires were distributed in all the four schools. Of these 367 questionnaires, 358 were kept for analysis, and 9 discarded because they were mostly incomplete. 54.5% of the respondents were male; 45.5% female. Considering the relatively small variance in age, participants were divided into only two groups: 12–15 years old (77.7%) and 16–21 years old (22.3%). Mean age and variance across the entire sample was 14.44 ± 1.61years old. 33.4% of students were Dutch and 66.2% were Belgian. All participants had a level of schooling below or equal to the 12th grade. In terms of household composition, 19.2% of the students lived under the guardianship of a single parent and 80.8% under the guardianship of a couple. 44.1 % of the participants lived in urban areas and 55.9% in rural areas. Analysis of the different housing types showed that 7.6% of the children lived in apartments, 42.2% in semi-detached houses, and 50.1% in detached houses. 92.1% reported having a garden, while 7.9% did not. The frequency of visits to zoos and aquariums was predominantly ‘once every two years or less’ (31.7%), then ‘once per year’ (27.8%), followed by ‘once every six months’ (22.1%), ‘never’ (15.6%), and lastly ‘once a month or more’ (2.8%). In the sample of students studied, 0.6% were vegetarian, 5.6% ate meat once a week or less, 19% ate meat two or three days a week, 43% four to six days a week, and 31.9% ate meat every day. Regarding pet ownership, 77% replied that they had a pet, while 23% did not. 

### 3.2. The AIS Score

The total AIS score for participants in the study was on average 158.61 out of 215 (see Table 1). Students scored relatively high (high score here means less acceptability) on variables related to: ‘deprive animal welfare’ (22.82 out of 25), ‘harm animals for environment’ (23.96 out of 30), ‘harm animals for social issues’ (24.00 out of 30), and ‘killing animals’ (18.74 out of 25).

A multiple regression analysis was run to find the factors most predominantly influencing AIS questionnaire scores (significance value *p* < 0.05). Pet ownership was an important determinant, with students who owned any pet scoring 5.41 points higher on average, compared to those who did not. However, only owning a pet dog is significant. AIS scores were 6.30 points higher for students who were dog owners, indicating that owning a dog may strongly influence the AIS scores. Test results also showed that the country of origin influences AIS scores: Belgian students scored 9.81 points higher than Dutch students. Gender was another important determinant in the AIS score; with females scoring 8.54 points higher than males. Lastly, eating meat also influenced total AIS scores (Table 2). 

#### 3.2.1. Age Groups

There was no significant difference between the two age groups in terms of their total score on the AIS questionnaire (U = 5612.500, N (12–15 years old) = 194, N (16–21 years old) = 63, *p* = 0.331). This implies that age had no influence on responses. However, the Mann-Whitney U-test did show a significant difference between 12–15 years old participants and 16–21 years old participants in the scoring results for one variable: ‘Use of animals’ (U = 7823.500, *p* = 0.007). The median score in this specific section of the questionnaire was significantly higher for 12–15 year olds (Mdn = 16.00) than for 16–21 year olds (Mdn = 15.00). 

#### 3.2.2. Gender 

Results indicate a significant difference in the total score for the AIS between the two genders (U = 5858.000, N (males) = 150, N (females) = 107, *p* = 0.000). Females had a higher median total score for the AIS questionnaire (Mdn = 165.00) than males (Mdn = 156.00) (Table 4). This suggests that female students scored higher overall on the AIS questionnaire. 

A statistically significant difference between the two genders was observed for all variables, except two: ‘Changes in animal’s genotypes’ (U = 11646.000, *p* = 0.988) and ‘Animal integrity destruction’ (U = 13944.000, *p* = 0.801). Females scored significantly higher than males for the following variables: ‘Use of animals’ (U = 11680.500, *p* = 0.017), ‘Killing animals’ (U = 11704.500, *p* = 0.009), ‘Deprive animal welfare’ (U = 11522.000, *p* = 0.003), ‘Experimentation on animals’ (U = 9889.500, *p* = 0.000), ‘Harm animals for environment’ (U = 7954.500, *p* = 0.000) and ‘Harm animals for social issues’ (U = 9102.000, *p* = 0.000). For all variables, median values were higher for female participants. These results were in accordance with results obtained from the analysis of the total AIS score. 

#### 3.2.3. Nationality 

Test results for the nationality variable indicate a significant difference between questionnaire scores for variables ‘Use of animals’ (U = 9561.500, *p* = 0.000), ‘Animal integrity destruction’ (U = 6709.00, *p* = 0.000), ‘Killing animals’ (U = 9448.000, *p* = 0.000), ‘Deprive animal welfare’ (U = 10113.500, *p* = 0.000), ‘Experimentation on animals’ (U = 10346.500, *p* = 0.023), ‘Changes in animal’s genotypes’ (U = 6544.000, *p* = 0.000), ‘Harm animals for environment’ (U = 9807.500, *p* = 0.036). The only non-significant exception was the following variable: ‘Harm animals for social issues’ (U = 11475.500, *p* = 0.392). 

This is in alignment with results for the total score of the AIS questionnaire (see below), which indicate a significant difference between Belgian and Dutch respondents (U = 5069.500, N (Belgian) = 157, N (Dutch) = 100, *p* = 0.000). The total median score was also higher for Belgian participants (Mdn = 164.00) than for Dutch participants (Mdn = 152.50). 

#### 3.2.4. Household 

The total score of the AIS questionnaire was not found to be significantly different between those students living with a single parent, and those living with a couple (U = 3722.00, *p* = 0.250). However, there were significant differences in scores for the following separate sections of the questionnaire: ‘Use of animals’ (U = 5806.000, *p* = 0.036, Mdn = 17.00) and ‘Animal integrity destruction’ (U = 5703.500, *p* = 0.006, Mdn = 22.00), with higher medians attributed to students living with a single parent.

#### 3.2.5. Residence Area

No significant difference was found between the total scores for students who lived in rural areas and those who lived in urban areas (U = 6764.500, *p* = 0.825), nor between any of the separate sections of the questionnaire (*p* < 0.05 in all cases). 

#### 3.2.6. House Type

No significant difference was found between the three groups of participants for residence (either: apartment, semi-detached house, or detached house). All p-values were higher than the threshold value of 0.05, which suggests that the type of house wherein students lived did not influence their AIS questionnaire score. 

#### 3.2.7. Owning a Garden

Ownership of, or access to a garden did not influence total scores on the AIS questionnaire (U = 2215.00, *p* = 0.955). Furthermore, no significant difference in scores was found in any individual section of the questionnaire. 

#### 3.2.8. Zoo/Aquarium Visiting Frequency

Equality of variance was assumed for all variables except for: ‘Harm animals for social issues’. A significant difference in total scores was found between students, based on how often they visited a zoo or an aquarium (χ2 (2) = 9.624, *p* = 0.047). Significant differences were also noticed in the following separate variables of the questionnaire: ‘Harm animals for environment’ (χ2 (2) = 14.823 *p* = 0.005), ‘Deprive animal welfare’ (χ2 (2) = 10.936, *p* = 0.027). A post-hoc analysis of variables showed that students who never visited zoos and/or aquariums had lower total AIS scores than those who did. 

#### 3.2.9. Meat-Eating Frequency 

Equality of variance was assumed for all variables except: ‘killing animals’ and ‘experimentation on animals’. There was no significant difference in total scores between students who ate meat and those who did not (χ2 (2) = 8.825, *p* = 0.066). There was however a significant difference for the ‘Harm animals for social issues’ variable of the questionnaire (χ2 (2) = 3.862, *p* = 0.425). 

#### 3.2.10. Pet Ownership

Data analysis of the AIS questionnaire variables: ‘experimentation on animals’ (U = 6587.000, *p* = 0.000), ‘Deprive animal welfare’ (U = 8299.000, *p* = 0.021), and ‘killing animals’ (U = 7792.000, *p* = 0.003) show pet owners scored significantly higher than non-pet owners. The total AIS score was also significantly higher (U = 3989.000, *p* = 0.000, N (pet owners) = 198, N (non-pet owners) = 58) for pet owners. 

#### 3.2.11. Religious Outlooks 

Results indicate that there was no significant difference in the total score in replies to the question: ‘is religion important in your life?’ (U = 5942.500, N (yes) = 62, N (no) = 195, *p* = 0.841). Whilst analysis of the total score for the AIS questionnaire did not show any distinction between students who considered religion important and those who did not, there were significant differences in terms of individual sections of the questionnaire. Significant differences only occurred for the following variables: ‘Animal integrity destruction’ (U = 9626.500, *p* = 0.008) and ‘Killing animals’ (U = 9928.500, *p* = 0.044), with higher median scores for those students who replied religion was important in their lives. 

### 3.3. The AAS Score

The total AAS score for participants in the study was on average 67.94 out of 100 (see Table 3). The highest scores were observed for the following variables: ‘it is morally wrong to hunt wild animals just for sport (4.02 out of 5), ‘wild animals such as mink and raccoons should not be trapped and their skins made into fur coats’ (4.06 out of 5), ‘the slaughter of whales and dolphins should be immediately stopped even if it means some people will be put out of work’ (4.11 out of 5), and ‘breeding animals for their skins is a legitimate use of animals’ (4.01 out of 5). 

To summarise, results from multiple regression analysis showed that AAS questionnaire scores were mostly influenced by: sex, as females scored 7.85 points higher than males on the questionnaire; meat-eating frequency, as those who ate meat less frequently scored 7.85 points higher than others; pet ownership, as pet owners scored 5.55 points higher than non-pet owners; and nationality, as Belgian students scored 5.13 points higher than Dutch students. Finally, the residence type students lived in influenced AAS total scores, yet as seen with the Kruskal–Wallis test, the religion (Christianity) was not significantly influence respondents’ attitudes toward animals (Table 4). 

#### 3.3.1. Age 

There was no significant difference between the total AAS scores, for students 12–15 years old and those who were 16–21 years old (U = 8759.500, N (10–15 years old) = 245, N (16–21 years old) = 76, *p* = 0.436). 

#### 3.3.2. Gender 

Female students had significantly higher total AAS scores than males (U = 7522.500, N (females) = 146, N (males) = 175, *p* = 0.000). Higher medians for females were also found in the individual AAS questionnaire questions. 

#### 3.3.3. Nationality 

The total AAS score was significantly higher for Belgian students than Dutch students (U = 8177.000, N (Dutch students) = 114, N (Belgian students) = 209, *p* = 0.000). Likewise, medians for the individual AAS questions were in general, higher for Belgian students. 

#### 3.3.4. Household 

Students who lived with a single parent scored significantly higher on the total AAS score than those who lived with a couple (U = 5419.000, N (students living with single parents) = 58, N (students living with parents forming a couple) = 236, *p* = 0.036). Regarding the individual AAS questions, medians were generally higher for students living with a single parent. 

#### 3.3.5. Residence Area 

Place of residence (either urban or rural area) did not influence total AAS scores in any significant way, as shown in Table 4 (U = 10810.500, N (students living in urban areas) = 130, N (students living in rural areas) = 168, *p* = 0.882). 

#### 3.3.6. House Type 

Living in either an apartment, a semi-detached house or a detached house did not significantly influence the total AAS score (χ2 (2) = 0.401, N (students living in an apartment) = 22, N (students living in a semi-detached house = 131, N (students living in a detached house) = 157, (*p* = 0.818) of students. 

#### 3.3.7. Owning a Garden 

There was no significant difference in the total AAS scores between students who had a garden and those who did not (U = 3455.500, N (students who have a garden) = 297, N (students who do not have a garden) = 24, *p* = 0.804). 

#### 3.3.8. Zoo/Aquarium Visiting Frequency 

There was a significant difference in the total AAS score for students depending on how often they visited zoos and/or aquariums (χ2 (2) = 21.989, N (once a month or more) = 9, N (once every six months) = 75, N (once every year) = 88, N (once every two years or less) = 101, N (never) = 47, *p* = 0.000). A post-hoc analysis revealed that those who never went to a zoo and/or aquarium scored significantly lower than others on the AAS questionnaire. 

#### 3.3.9. Meat-Eating Frequency 

There was a significant difference in total AAS score (χ2 (2) = 15.420, N (I do not eat meat, I am vegetarian/vegan) = 2, N (once a week or less) = 17, N (2–3 days a week) = 63, N (4–6 days a week) = 134, N (everyday) = 96, *p* = 0.004). A post hoc analysis revealed higher scores for those who ate meat less frequently (once a week or less) than others. 

#### 3.3.10. Pet Ownership

A significant difference was found between students who owned a pet and those who did not, in their total AAS score. Those who had pets scored significantly higher (U = 6359.500, N (pet owners) = 250, N (non-pet owners) = 73, *p* = 0.000). Overall, medians were also higher for pet owners. 

#### 3.3.11. Religious Affiliation

Religious affiliation had no significant influence on total AAS scores from students as shown in Table 4 (U = 10146.000, N (religion/spirituality is important) = 92, N (religion/spirituality is not important) = 232, *p* = 0.000). 

## 4. Discussion and Conclusion 

Results from the study revealed several strong correlates for young adults’ attitudes towards animals. The most important factors identified here were: gender, nationality, zoo/aquarium visiting frequency and pet ownership. Similarly to results reported by previous research [5,12,13], students’ attitudes towards animals measured by both AIS and AAS were found to be relatively positive. 

Female respondents scored higher on both AAS and AIS than their male counterparts. Girls showed more concern for animals specifically in categories where the welfare and life of the animal was compromised (e.g. ‘killing animals’, ‘experimentation on animals’, ‘Harm animals for environment’). There were no differences between the two genders for items which involved animals being treated to improve their appearance or productivity (‘changes in animals’ genotypes’ and ‘animal integrity destruction’). The present results confirm findings of previous studies on gender differences identifying a prevalent female inclination for animal well-being and nurturing [3,6,10,14,15,16,17]. 

Results also showed that Belgian students scored significantly higher in the attitudes questionnaires for most items in contrast to Dutch students. Previous studies have revealed fairly similar attitudes from citizens in both countries, in contrast to the present study. In his study on the welfare of pets in commerce, Dewar found that both Dutch and Belgian respondents were in favour of improving animal welfare in their respective countries. Additionally, in a study investigating the use of animals in society, European students expressed more concern for animal well-being than Asian students [12,14]. However, more closely aligned to the present study were results from Pifer, Kinya Shimizu and Pifer [15]. These authors found that Belgian respondents expressed more opposition towards animal research than their Dutch counterparts. The two countries do both in fact express concern for animal well-being; however Belgian respondents may be somewhat more passionate about these issues than Dutch students. 

The present results also suggest a link between visits to the zoo/aquarium and positive attitudes towards animals. Young adults who reported that they never visited zoos or aquariums had lower AIS and AAS scores than the others. These findings support claims that zoos can fulfil more roles than mere entertainment, by encouraging learning experiences, a sense of connection towards wild animals, and focusing people’s attention on conservation issues [18,19]. However, Tunnicliffe, et al. [20] warn that these connections can only lead to a better-educated public if zoos integrate the experience with follow up discussions and leave space for reflection. Special attention should also be paid to less popular animals (e.g. bats, spiders etc.) to increase awareness on the vital role of biodiversity. 

Pet ownership was another significant correlate in determining students’ attitudes. Analysis showed that students who owned a pet scored higher on the questionnaires and expressed greater concern for animal welfare than students without a pet. These outcomes are consistent with Prokop and Tunnicliffe [21] previous findings on the relation between pet ownership and positive attitudes towards other animals. However research in this area has not yet shown consistent results and theories oscillate between whether pet ownership has a relationship to young adults’ attitudes towards animals [22]; for example, how such ownership might correlate with attitudes towards less popular animal species [23]. 

Regarding diet, significant differences were only observed in response scores for the AAS questionnaire. Those who ate meat once a week or less had higher scores than those who ate meat more frequently. The expression of higher concern for animal welfare from those who report to eat very little to no meat may be explained through the same line of thought found in Amato and Partridge [24] work on vegetarians. Here, the authors reported that a majority of vegetarians had made their dietary choice for ethical beliefs in animal rights. Another study on vegetarian girls revealed that most also made their choice on an ethical basis, and as an effort to reduce animal suffering [25]. Furthermore, these results can also be interpreted in a similar fashion to those of Hagelin, Carlsson and Hau [17] who report that concern for animals killed for food can also be extended to a concern for animal well-being in other domains such as animal research. Finally, a short comment must be made on the relatively small number of self-reported vegetarians in the present study. Most students ate meat at least once a week; however, in those responses a few had added that they wished to be vegetarian, but their parents wouldn’t allow it. The results are therefore not entirely reflective of the dietary choices of all students. 

Another important variable was household composition. Students living with a single parent demonstrated more concern for animal welfare in the questionnaire than those who lived with two parents. Perhaps these differences can be explained by Albert and Bulcroft [26] work on pet owners, who wrote that, ‘‘Attachment to pets is highest among never married, divorced, widowed, and remarried people, childless couples, newlyweds, and empty-nesters. Never married, divorced, and remarried people, and people without children present, are also most likely to anthropomorphize their pets.’’ The young adults’ single parents in the present study fall under the category of ‘never married’ or ‘divorced’. As a parents’ behaviours influence that of their child [27], it may be possible that these young adults adopted similar attitudes. 

No differences were found between young adults who lived in urban areas and those who lived in rural areas, as has also been found in China [8]. This is despite observations that urban and rural citizens have different opportunities to interact with animals, as is reflected in the finding of greater knowledge about animals in rural residents compared to city dwellers [3]. Perhaps one reason that the present study found no differences between urban and rural residents is because the “urban” areas reflected in the present demographic were actually somewhat rural; small towns in close proximity to surrounding rural environments. In further support, neither the type of residence nor garden access correlated with attitudes in the present study.

Religion, more specifically Christianity, showed a weak relationship to young adults’ attitudes towards animals but only for a few particular questionnaire items rather than to overall scores. Items on the AIS that asked students for how acceptable they found the killing of animals or the destruction of their integrity correlated with higher values reported for the importance of religion in a respondent’s life.

Finally, age did not significantly correlate with attitudes. Because others have reported that significant changes in attitudes towards animals occur throughout childhood [3], this finding was unexpected. However, variability in age in the present sample study group was small, and this may be why no relationship was found between age and attitude ratings.

As shown in the present study, pet ownership is usually associated with positive attitudes towards other animals [21,22]. It is important to note however that pet ownership is not necessarily an end-all contributing factor to more positive attitudes. Although there is a relative correlation between pet ownership and more positive attitudes towards other animals, there is no guarantee that this attitude will extend to all animal species. The popularity, familiarity, biophilia (attraction), and the types of emotions that an animal species triggers can greatly influence the protection and welfare it receives from humans [28]. Likewise, Vining [29] stresses that emotion is at the heart of the actions or inactions of humans in terms of the respect and protection they provide animals. Furthermore, what arguably matters more is the quality of the relationship between young adults and their pets, or other animals in general. A study about animal abuse showed that fear of animals was a considerable determinant of negative attitudes (cruelty, apathy etc.) [6]. This again highlights the importance of engaging in meaningful connections with animals.

Another positive correlated factor to positive attitudes are visits to zoos or aquariums. Young adults gain knowledge and significant appreciation for the environment and its different species, when learning outside the classroom setting, in direct contact with nature and wildlife [30]). Informal educational settings such as zoos and aquariums should work to ensure exposure of their visitors to less popular animal species (e.g., pests, predators), in order to help students to understand the importance of each species in the ecosystem [21,28]. A commitment to education is a common element in the mission statements of contemporary zoos; such institutions can make substantive contributions towards improving public understanding of and appreciation for an animal’s specific role in the ecosystem and thus enhance positive attitudes towards that animal [28].

Lastly, the present study found that those who reported eating meat less frequently (once a week or less) also had more positive attitudes towards animals and their welfare as measured in one of the scales used (the AAS). People who opt to eat little or no meat may do so for many different reasons, including reasons having to do with health, economics, and/or an interest in reducing the ecological impact of meat production, as well as for reasons that stem from a moral objection to consuming animals [31]. It should be noted that in the present study, the number of respondents stating that they ate meat only rarely was small (only about 5% of the total number of respondents); nonetheless the significant difference between this group and others in the study suggests that moral convictions that affect dietary choice may also correlate with moral convictions about the humane treatment of animals.

As this paper has shown, a variety of variables correlate with young people’s attitudes towards animals and their welfare. A better understanding of the causes of these correlations and the development of these variables over the lifetime of a child may help us to better structure the kinds of experiences that promote empathy and concern for all living things. 

## Figures and Tables

**Table 1:** Descriptive statistics: total Animal Issue Scale (AIS) and individual variables mean scores.

	Minimum	Maximum	Mean	SD
Total AIS	79	206	158.61	18.76
Use of animals (score: 5–25)	6	24	15.36	3.21
Animal integrity destruction (score: 6–30)	12	30	21.05	3.16
Killing animals (score: 5–25)	9	25	18.74	2.79
Deprive animal welfare (score: 5–25)	5	25	22.82	2.67
Experimentation on animals (score: 5–25)	5	59	16.40	4.38
Changes in animals’ genotypes (score: 5–25)	5	25	17.00	4.13
Harm animals for environment (score: 6–30)	0	30	23.96	3.96
Harm animals for social issues (score: 6–30)	0	30	24.00	3.69

**Table 2:** Correlations between independent questionnaire variables and the total AIS score.

Y: Total AIS Score	Unstandardized Coefficients		Standardized Coefficients	t	*p*
	B	Std. Error	Beta		
(Constant)	160.80	9.03		17.81	<0.001
X_1_: Do you own a pet?	−5.41	3.16	−0.13	−1.71	0.088
X_2_: In what country do you live?	9.81	2.38	0.27	4.13	<0.001
X_3_: What is your sex?	8.54	2.28	0.24	3.74	<0.001
X_4_: How often do you eat meat (including fish) every week?	−3.34	1.35	−0.16	−2.47	0.014
X_5_: What pet (s) do you have? Dog (s)	−6.30	2.64	−0.18	−2.39	0.018

**Table 3:** Descriptive statistics: total Animal Attitude Scale (AAS) and individual variables mean scores.

	Minimum	Maximum	Mean	SD
Total AAS	37	99	67.94	10.33
1. It is morally wrong to hunt wild animals just for sport.	1	5	4.02	1.18
2. I do not think that there is anything wrong with using animal in medical research.	1	5	3.32	1.66
3. There should be extremely stiff penalties including jail sentences for people who participate in cock-fighting.	1	5	3.56	1.11
4. Wild animals, such as mink and raccoons, should not be trapped and their skins made into fur coats.	1	5	4.06	1.16
5. There is nothing morally wrong with hunting wild animals for food or a better living for poor people.	1	5	2.55	1.07
6. I think people who object to raising animals for meat are too sentimental.	1	5	2.99	1.11
7. Much of the scientific research done with animals is unnecessary and cruel.	1	5	3.21	1.08
8. I think it is perfectly acceptable for cattle and dogs to be raised for human consumption.	1	5	3.08	1.27
9. Basically, humans have the right to use animals as we see fit.	1	5	3.73	1.22
10. The slaughter of whales and dolphins should be immediately stopped even if it means some people will be put out of work.	1	5	4.11	1.13
11. I sometimes get upset when I see wild animals in cages at zoos.	1	5	2.61	1.18
12. In general, I think that human economic gain is more important than setting aside more land for wildlife.	1	5	3.56	1.16
13. Too much fuss is made over the welfare of animals these days when there are many human problems that need to be solved.	1	5	3.16	1.21
14. Breeding animals for their skins is a legitimate use of animals.	1	5	4.01	1.11
15. Some aspects of biology can only be learned through dissecting preserved animals, such as cats.	1	5	3.15	1.22
16. Continued research with animals will be necessary if we are to ever conquer diseases such as cancer, heart disease and AIDS.	1	5	2.64	1.06
17. It is unethical to breed purebred dogs for pets when millions of dogs are killed in animal shelters each year.	1	5	3.48	1.05
18. The production of inexpensive meat, eggs, and dairy products justifies maintaining animals under crowded conditions.	1	5	3.04	1.08
19. The use of animals, such as rabbits, for testing the safety of cosmetics and household products is unnecessary and should be stopped.	1	5	3.82	1.20
20. The use of animals in rodeos and circuses is cruel.	1	5	3.78	1.20

**Table 4:** Most prevalent correlations between independent questionnaire variables and the total AAS score.

Y: Attitudes towards animals	Unstandardized Coefficients		Standardized Coefficients	t	*p*
	B	Std. Error	Beta		
(Constant)	74.07	5.77		12.84	<0.001
X_1_: What is your sex?	7.85	1.12	0.38	7.00	<0.001
X_2_: How often do you eat meat (including fish) every week?	−2.14	0.67	−0.16	−3.18	0.002
X_3_: What is your main source of inspiration? Christianity	−2.73	1.46	−0.10	−1.87	0.063
X_4_: Do you own a pet?	−5.68	1.36	−0.23	−4.18	<0.001
X_5_: In what country do you live?	4.82	1.25	0.23	3.86	<0.001
X_6_: In what sort of house do you live?	−2.11	0.97	−0.12	−2.18	0.030
